# Signatures and Prognostic Values of N6-methyladenosine (m6A) - related Immune Genes in Bladder Cancer

**DOI:** 10.1080/21655979.2021.1937910

**Published:** 2021-06-11

**Authors:** Gaoteng Lin, Jianwei Zhang, Yuqi Wu, Shimiao Zhu, Gang Li

**Affiliations:** a Department of Urology, Tianjin Institute of Urology, The Second Hospital of Tianjin Medical University, Tianjin, P.R. China; b Department of Urology, Tianjin Baodi Hospital, Baodi Clinical College of Tianjin Medical University, Tianjin, China

**Keywords:** Bladder cancer, m6A modification, tumor microenvironment, prognostic prediction

## Abstract

In recent years, genes associated with N6-methyladenosine (m6A) modification were found to participate in modulation of multiple tumor biological processes. Concomitantly, the significantly complicated dual effects of tumor microenvironment have been observed on cancer progression. The present study aims to investigate m6A-related immune genes (m6AIGs) for their signatures and prognostic values in bladder cancer (BC). Out of 2856 differentially expressed genes (DEGs) of BC, a total of 85 genes were obtained following intersection of DEGs, immune genes and m6A-related genes. The results of multivariate Cox regression analysis illustrated four genes (BGN, GRK5, IL32, and SREBF1) were significantly associated with the prognosis of BC patients. The BC samples were divided into two types based on the consensus clustering, and the principal component analysis demonstrated a separation between them. It was found that high expression of BGN and GRK5 were linked with advanced T and N stage, and the expression of SREBF1 in early T stage was higher than that in advanced T stage. Subsequently, the nomogram to predict 3- and 5-year survival probability of BC patients was developed and calibrated. GSEA analysis for risk subgroups showed WNT and TGF-beta signaling pathways were involved in regulation of BC progression in high risk level group. In the low risk level group, cytosolic DNA-Sensing cGAS-STING and RIG-I-like receptors signaling pathways were found to be correlated with BC development. These findings provide a novel insight on studies for BC progression.

## Graphical abstract

**Figure:**
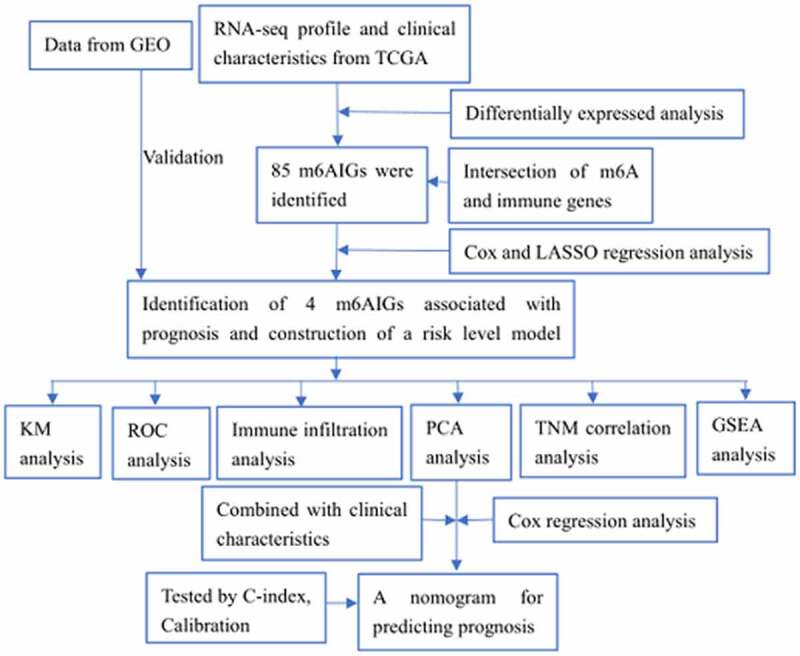

## Introduction

Bladder cancer (BC) is a disease with high incidence and mortality, ranking 13th in respect of the number of deaths, the new cases of BC reach approximately 550,000 and the number of deaths reaches 200,000 in the world per year [1]. Urothelial BC is the major subtype of BC. About 75% of patients with BC are diagnosed as non-muscle-invasive disease (NMIBC), and because of the risk of recurrence and progression, a timely intervention and active surveillance are extraordinary necessary [2,3]. The remaining 25% are advanced cancer called muscle-invasive disease (MIBC), treated with operation centered comprehensive therapies [3,4], but recurrence is the serious problem with MIBC after radical cystectomy [5]. Identifying biomarkers related with the prognosis and improving the accuracy of prediction of recurrence and progression are essential for the management and treatment of patients with BC.

New evidences have showed that bladder inflammatory disease increased the risk of developing cancer [6], and studies on tumor microenvironment (TME) [7,8] demonstrated that the tumor infiltrating immune cells (TIICs) were closely associated with the growth and progression, immune escape, infiltrated metastasis, recurrence and clinical outcomes in varied tumors. Neutrophils, mast cells, eosinophils, NK cells, B cells, some subpopulation of T cells and M2 phenotype of tumor-associated macrophages were capable to promote angiogenesis by diverse mediators and signaling pathways, leading to tumor growth and progression [9]. The loss of functions of natural killer cells (NK) and CD8 + T cells caused from suppression by tumor-associated macrophages and neutrophils through production and expression of various factors contributed to immune escape following metastasis [10]. The majority of TIICs had a clear effect on clinical events, and due to the variety and abundance of TIICs, the impact on clinical outcomes varied from tumor types [8]. Curiel et al. demonstrated a correlation between regulatory T cells and the poor survival in ovarian cancer patients [11], in contrast, Winerdal et al. suggested regulatory T cells with FOXP3 expression prolonged the overall survival of BC patients [12].

More than 100 types of post-transcriptional modification on RNA have been identified [13]. Apart from the modification of 5ʹ cap and the 3ʹ poly (A) tail – already known, eukaryotic RNA also features ubiquitous and dynamic N6-methyladenosine (m6A) internal modification [14]. The dynamic modifications of RNA m6A was involved in the installation, recognition and removal [13], executed by methyltransferases (such as METTL3, termed as ‘Writers’), m6A-specific binding proteins (such as YTHDF1, termed as ‘Readers’) and demethylases (such as FTO, termed as ‘Erasers’) respectively [15]. RNA with m6A modification gains the functions as metabolism regulation, structural changes, affecting maturation, facilitating decay and cell function shaped, etc, to enable the post-transcriptional gene regulation [15]. Further evidences suggested tumorigenesis, progression and metastasis on tumors were highly correlated with changes of the level of gene expression resulting from dysregulation of m6A modification [16,17]. Increasing global m6A RNA modification via FTO activity inhibited by R-2-hydroxyglutarate (R-2 HG) attenuated the stability of MYC/CEBPA transcripts, suppressing tumor signaling pathway for anti-leukemic functions [16]. METTL3 facilitated the proliferation of BC through -acceleration of maturation of pri-miR 221/222 in an m6A-dependent manner to decrease PTEN expression, and METTL3 was also capable of modulating the AFF4/NF-κB/MYC signaling pathway in an m6A-dependent manner, giving rise to progression of BC [17]. In ovarian cancer, YTHDF1 could bind to EIF3C mRNA with m6A modification to elevate translation of EIF3C, thereby leading to tumorigenesis and metastasis [18]. Some studies showed m6A modification regulators could serve as tumor suppressors. METTL3/14 could inhibit the growth and self-renewal of glioblastoma stem cell through influence of m6A enrichment and transcription [19]. In hepatocellular carcinoma, METTL14 served as an inhibitor on metastasis by regulating microRNA 126 in an m6A-dependent manner [20].

The most common chemical modification in eukaryotic cells enables us to concentrate on the signatures of post-transcriptional immune genes of m6A modification for identifying potential biomarkers in BC. Since m6A modification and tumor microenvironment play an important role in regulating tumor progression, the impact of m6AIGs on tumor behaviors are of interest. It is therefore very important to screen prognosis-associated m6AIGs in BC. However, no m6AIGs were identified in BC. The present study aims to identify m6A-related immune genes associated with prognosis, construct a risk level model based on m6AIGs, analyze the influence of m6AIGs on tumor progression and patients’ prognosis, develop a robust and reliable nomogram to predict prognosis and investigate the potential regulatory pathways.

## Material and methods

### Acquisition and analysis of relevant dataset

The gene expression profile containing 414 tumor and 19 normal tissues of BC samples and the clinical characteristics of corresponding patients (n = 412), including age, gender, stage, grade, Tumor-Node-Metastasis (TNM) classification, were obtained from TCGA database (https://portal.gdc.cancer.gov/). Cohort with prognostic characteristics was gained from Gene Expression Omnibus (GEO) (GSE31684) (https://www.ncbi.nlm.nih.gov/geo/query/acc.cgi?acc=GSE31684) [21]. The missing or unknown values in the clinical datasets were excluded. The R package (‘DESeq2’) was utilized to perform differentially expressed analysis on the gene expression profile of BC in terms of the criteria of | log2-fold change | > 1 and false discovery rate (FDR) < 0.05 [22]. The immune gene dataset and the m6A-related gene dataset were downloaded from InnateDB (https://www.innatedb.com/) [23] and RMVar (http://rmvar.renlab.org/) [24] respectively. All data processing and drawing were accomplished with R software (version: 4.0.2). Analytical data with *p* < 0.05 was regarded as statistically significant.

### Screening m6A-related immune genes and assessing their signatures and prognostic values

The differentially expressed genes (DEGs), the immune genes and the m6A-related genes were intersected to obtain m6A-related immune genes (m6AIGs) in BC. The univariate Cox regression analysis was performed on m6AIGs to identify m6AIGs associated with the overall survival of BC patients. The result of univariate analysis was filtered with the Least Absolute Shrinkage and Selection Operator (LASSO) regression analysis, to avoid over fitting [25]. The multivariate Cox regression analysis was performed to finally determine independent m6AIGs affecting the prognosis. Then the risk score model was constructed based on the results of multivariate Cox regression, i.e. multiplying the expression values of m6AIGs with *P* < 0.05 by their coefficient in the model and then adding them together. The receiver operating characteristic (ROC) curve showed the performance of the risk score model for 3-and 5-year prediction. External cohort was used to validate the risk score model and the ability of prediction for ROC. The Kaplan–Meier (KM) survival analysis was performed on independent m6AIGs and the risk score level of BC patients.

### Expression in immune cells

The correlation of the screened m6AIGs expression with immune cells infiltration level was analyzed in the Tumor IMmune Estimation Resource (TIMER, https://cistrome.shinyapps.io/timer/) [26].

### Consensus clustering analysis and principal component analysis (PCA)

In order to investigate the classification of BC subtypes based on m6AIGs, the consensus clustering analysis was used to estimate the category amount and determine the optimum category amount [27]. The rationality of the clustering among samples was evaluated with the PCA, and the differential survival among the categories was compared with the KM survival analysis.

### Analysis of expression level of m6AIGs in different TNM stages

The expression level of screened m6AIGs in different TNM stage was analyzed to elucidate the influence on tumor progression.

### Combination of clinical characteristics and construction of a nomogram

The univariate and multivariate Cox regression analyses were performed on clinical characteristics, the risk score level and the classification, to investigate the impact of these factors on the overall survival of BC patients; and a nomogram in terms of these factors was developed to predict the 3- and 5-year prognosis. The accuracy of the model was estimated with the concordance index (C-index) and calibration curve. The heatmap displayed the association between screened m6AIGs and the clinical characteristics, the risk score level and the classification.

### Signaling analysis of screened m6AIGs

The potential function and regulatory signaling pathways for risk level subgroups in BC were investigated by Gene Set Enrichment Analysis (GSEA) [28].

### Statistical analysis

All statistical analyses and plots were performed in R software. Cox and LASSO regression analyses were carried out to screen independent prognosis-associated m6AIGs. KM survival analysis with log-rank test was used to assess prognostic values of the m6AIGs. Student’s t-test was performed to evaluate the expression level of m6AIGs in different TNM stage. The statistically significant threshold was *P* < 0.05.

## Results

The present study aims to identify m6A-related immune genes associated with prognosis in BC. Four prognosis-related m6AIGs were screened, based on which a risk score level model was constructed. The model was validated by external cohort. Then, the immune infiltration analysis, PCA analysis and TNM analysis were performed to assess the impact of the four m6AIGs on tumor behaviors. Combined with clinical characteristics, a nomogram for predicting prognosis was developed and calibrated. Functional analysis based on GSEA was conducted to investigate the potential signaling pathways influencing tumor progression.

### Processing data from TCGA to obtain m6A-related immune genes

**Figure 1.**
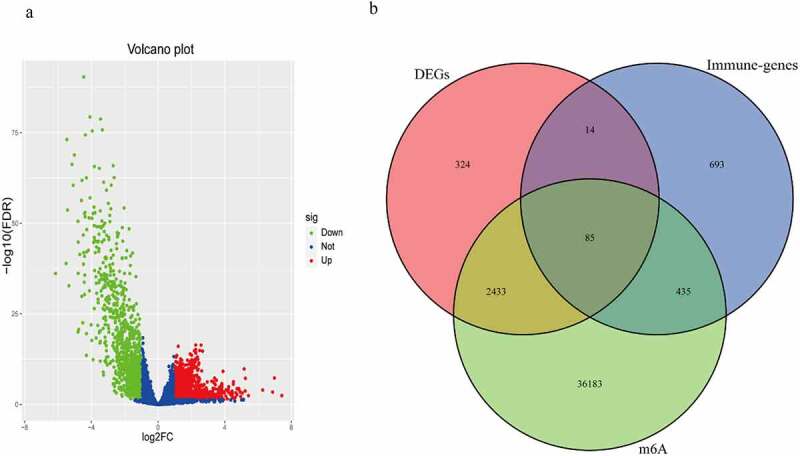
The m6A-related immune genes gained. (a) 2856 DEGs in BC were shown in volcano plot. The green, red, and blue dots mean downregulated and upregulated genes and no differential expression, respectively. (b) The Venn diagram shown the result of the intersection of the DEGs, immune genes and m6A-related genes

The total number of DEGs were 2856, as shown in Figure 1(a). The intersection was performed on DEGs, immune genes and m6A-related genes, to obtain 85 m6AIGs as shown in Figure 1(b). After clearing up the missing and unknown data, 373 clinical characteristics were obtained (Supplementary Table 1).

### Identifying m6AIGs associated with prognosis and investigating signatures

**Figure 2.**
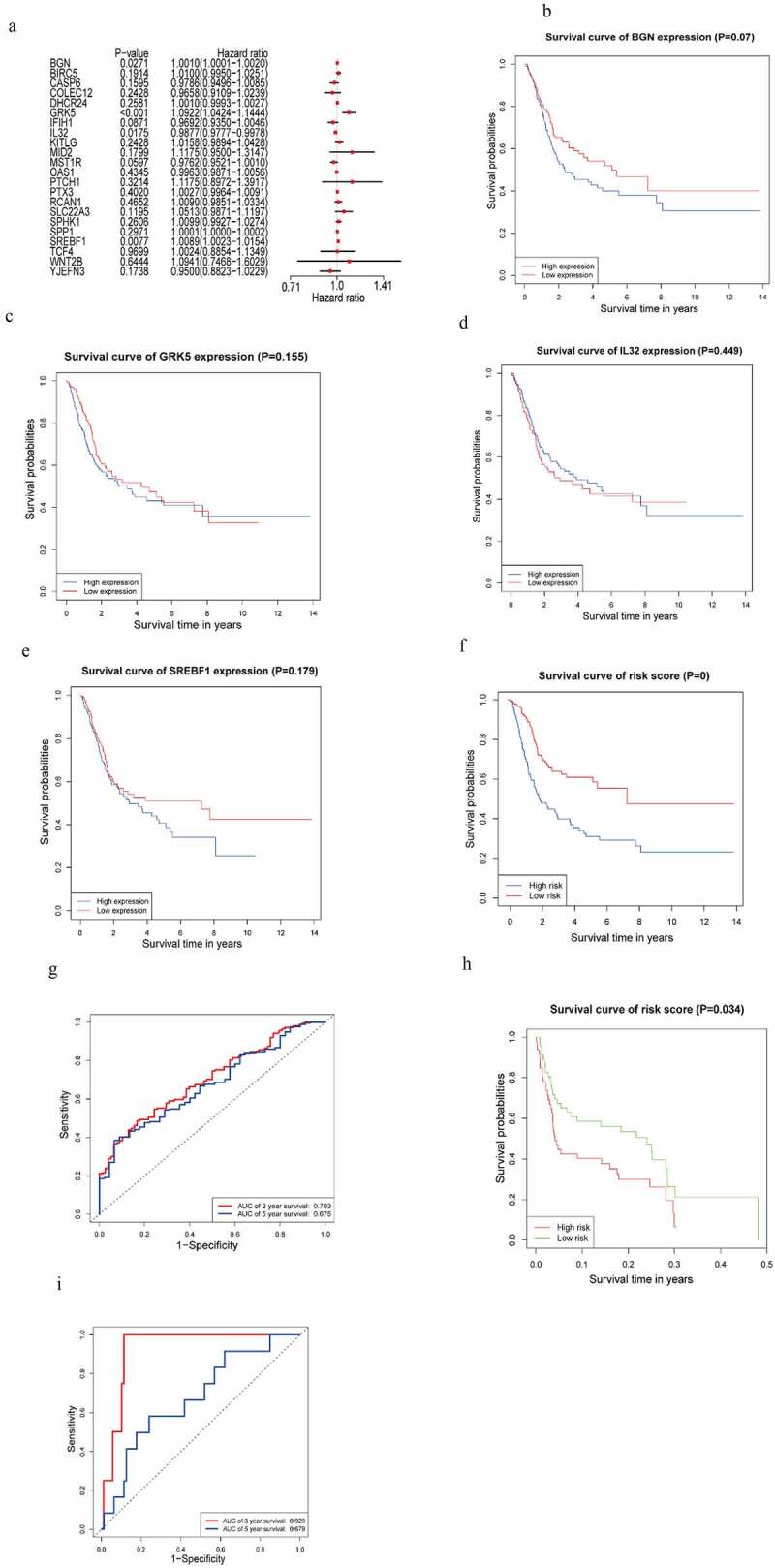
m6AIGs associated with prognosis and functional annotation. (a) Forest plot illustrated the result of multivariate Cox regression analysis for the 22 m6AIGs. (b–f) The survival analysis for the BGN, GRK5, IL32, SREBF1 and risk score level, respectively. (g) In TCGA cohort, ROC curve predicted the 3- and 5-year survival. (h) Risk score level model was developed within GEO cohort. (i) The area under ROC curve was calculated within GEO cohort

The outcome of the univariate Cox regression analysis on 85 m6AIGs was listed in Supplementary Table 2. A total of 28 m6AIGs were regarded as statistically significant. The LASSO analysis was carried out on m6AIGs (*P* < 0.05), obtaining 22 m6AIGs (Supplementary Figure S1a and 1b). The multivariate Cox regression analysis was performed on 22 m6AIGs, to obtain 4 m6AIGs, i.e. BGN (*P* = 0.0271, HR: 1.0010, 95% CI: 1.0001−1.0020), GRK5 (*P* < 0.001, HR: 1.0922, 95%CI: 1.0424−1.1444), IL32 (*P* = 0.0175, HR: 0.9877, 95%CI: 0.9777−0.9978) and SREBF1(*P* = 0.0077, HR: 1.0089, 95 CI%: 1.0023 − 1.0154), as independent prognostic factors, as shown in the forest plot in Figure 2(a). The prognostic model was developed on the basis of the 4 m6AIGs as follows: The risk score level model = (0.001035 × expression of BGN + 0.08818 × expression of GRK5 – 0.01236 × expression of IL32 + 0.008825 × expression of SREBF1). The median value of the model was 1.009, as the cutoff to classify samples into the low risk score level group and the high risk score level group. The KM survival analysis for each of the four m6AIGs did not show the differential survival in the light of log-rank test (Figure 2(b–e)), and the risk score level subgroups exhibited the survival difference (figure 2(f)). In TCGA cohort, the area under the ROC curve based on the risk score model for predicting the 3- and 5-year survival was 0.703 and 0.675, respectively (Figure 2(g)).

A risk score level model was also constructed based on the four m6AIGs within GEO cohort, to validate the performance of the model developed by TCGA cohort. The prognostic model based on GEO cohort was developed as follows: The risk score level model = (0.21 × expression of BGN + 0.44 × expression of GRK5 – 0.07 × expression of IL32 + 0.37 × expression of SREBF1). The median value of the model was 1.013. External cohort from GEO validated the survival difference between risk score level subgroups (Figure 2(h)). External cohort showed the area under the ROC curve for predicting the 3- and 5-year survival was 0.929 and 0.679 respectively (Figure 2(i)), showing the good performance of the risk score level model in predicting the prognosis.

### Four m6AIGs expression in immune cells

**Figure 3.**
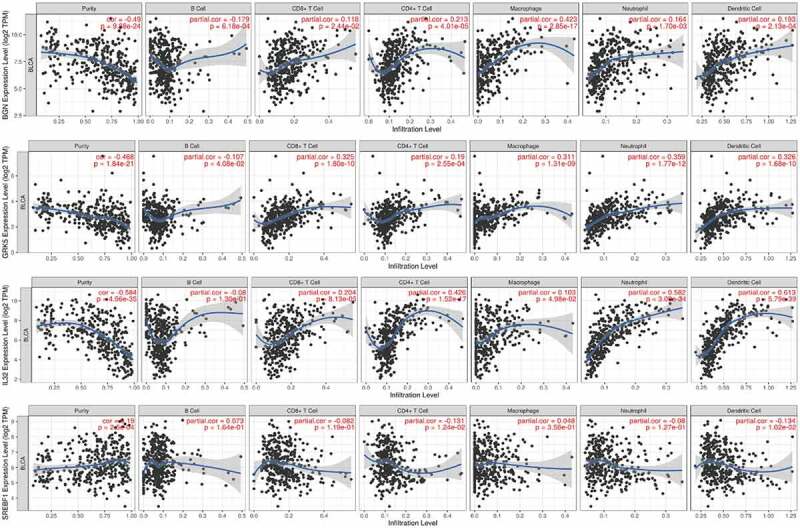
The correlation of the four m6AIGs expression with the tumor purity, B cell, CD8 + T cell, CD4 + T cell, Macrophage, Neutrophil and Dendritic cell

The TIMER analysis demonstrated that BGN expression was in highly negative correlation with B cell (*r* = −0.179, *P* = 6.18e−04), and positive association with infiltration of CD8 + T cell (*r* = 0.118, *P* = 2.44e-02), CD4 + T cell (*r* = 0.213, *P* = 4.01e-05), Macrophage (*r* = 0.423, *P* = 2.85e-17), Neutrophil (*r* = 0.164, *P* = 1.7e-03) and Dendritic cell (*r* = 0.193, *P* = 2.13e-04); that GRK5 expression was significantly in connection with B cell (*r* = −0.107, *P* = 4.08e-02), and was positively correlated with infiltrating CD8 + T cell (*r* = 0.325, *P* = 1.80e-10), CD4 + T cell (*r* = 0.19, *P* = 2.55e-04), Macrophage (*r* = 0.311, *P* = 1.31e-09), Neutrophil (*r* = 0.359, *P* = 1.77e-12) and Dendritic cell (*r* = 0.326, *P* = 1.68e-10); that IL32 expression was significantly positively associated with infiltration of CD8 + T cell (*r* = 0.204, *P* = 8.13e-05), CD4 + T cell (*r* = 0.426, *P* = 1.52e-17), Macrophage (*r* = 0.103, *P* = 4.98e-02), Neutrophil (*r* = 0.582, *P* = 3.09e-34) and Dendritic cell (*r* = 0.613, *P* = 5.79e-39); that SREBF1 expression was negatively correlated with infiltrating immune cells of CD4 + T cell (*r* = −0.131, *P* = 1.24e-02) and Dendritic cell (*r* = −0.134, *P* = 1.02e-02) (Figure 3).

### Classification for BC on the basis of four m6AIGs

**Figure 4.**
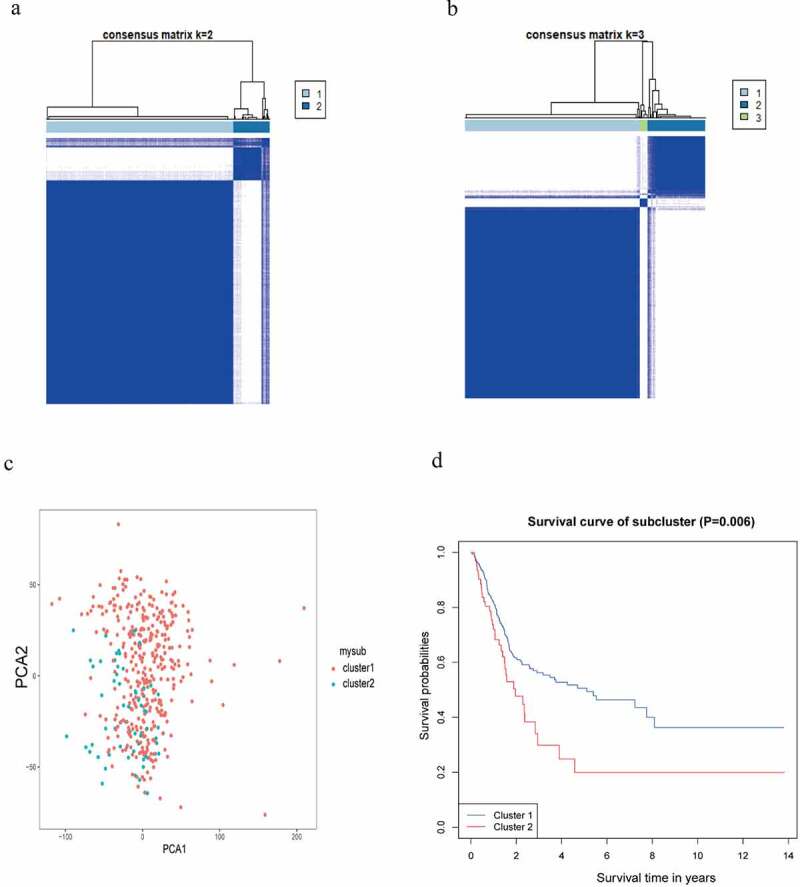
Identification of the category. (a, b) Determination of the optimum classification number. (c) The outcome of PCA. (d) KM survival analysis for BC samples in accordance with the two categories

The consensus clustering analysis demonstrated the optimum category in BC samples could be obtained when K = 2 (Figure 4(a-b)). When K took over other values, the classification was in such case as illustrated in Supplementary Figure S2. The PCA revealed a separation between the two classifications of BC samples (Figure 4(c)). The survival difference between the two classifications was analyzed by the KM survival analysis (Figure 4(d)).

### Correlation of expression of four m6AIGs with clinical traits of TNM stage

**Figure 5.**
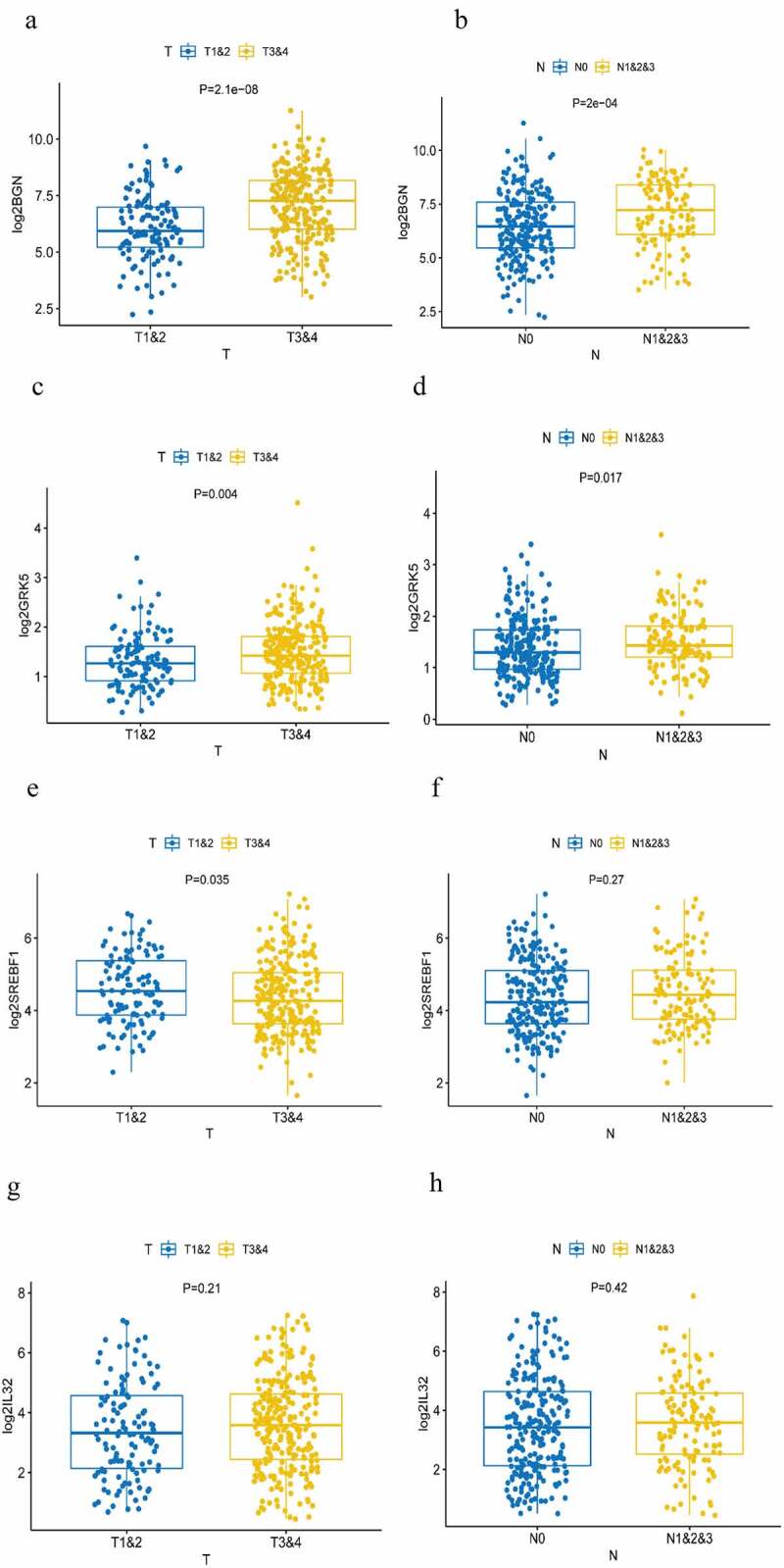
Analysis of TNM stage correlation . (a, b) The correlation of the expression of BGN with T and N staging. (c, d) The correlation of the expression of GRK5 with T and N staging. (e, f) The correlation of the expression of SREBF1 with T and N staging. (g, h) The correlation of the expression of IL32 with T and N staging

By comparing the expression level of the four m6AIGs in different TNM stages, high expression of BGN (Figure 5(a-b)) and GRK5 (Figure 5(c-d)) were found closely associated with advanced T and N stage, showing they were factors promoting tumor progression and lymph node metastasis. The expression of SREBF1 in early T stage was higher than that in advanced T stage (Figure 5(e)), suggesting it may be the factor facilitating tumorigenesis. There was no differential expression in N stage (figure 5(f). The expression of IL32 was not correlated with T and N stage (Figure 5(g-h)). There were no evidences demonstrating they were linked with M stage (Supplementary Figure S3a–3d).

### Analysis on clinical characteristics and prognostic prediction

**Figure 6.**
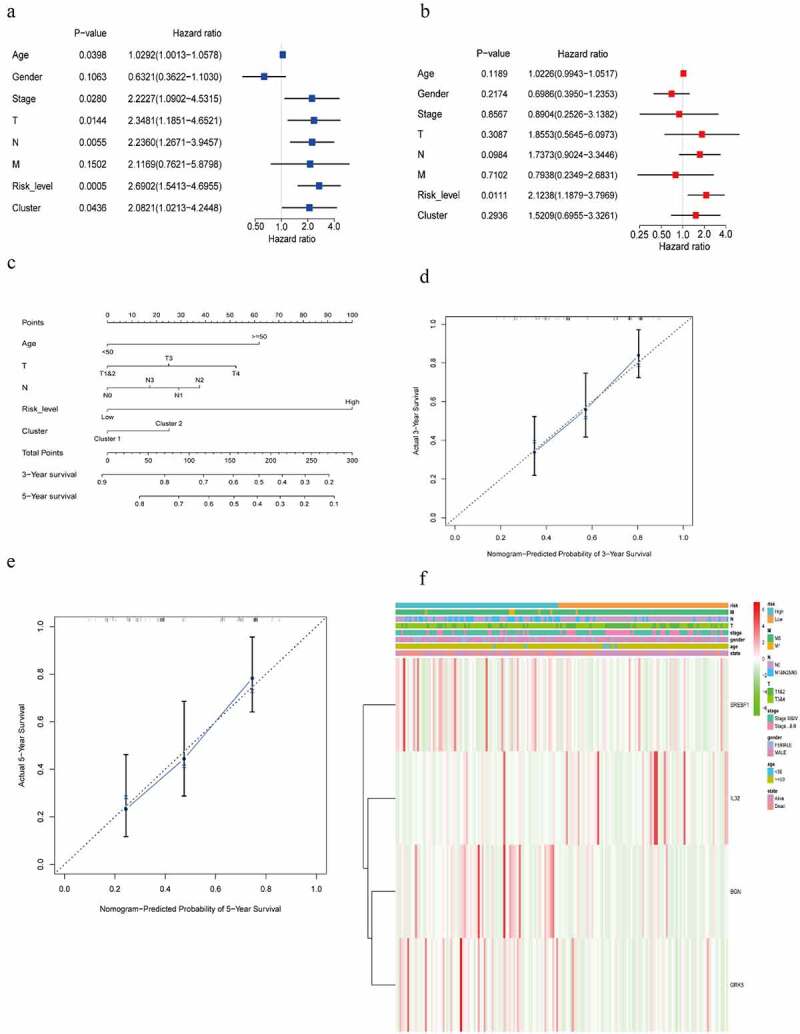
Screening the clinical characteristics and predicting prognosis. (a, b) Forest plot shown the result of the univariate and multivariate Cox regression analysis. (c) A nomogram based on age, T, N, risk score level, cluster, for predicting 3- and 5-year prognosis. (d, e) Calibration curve of nomogram for 3- and 5-year prediction. X-axis represents nomogram−predicted probability of overall survival and Y-axis stands for actual survival. The more the blue solid line fits the black dotted line, the better the prediction effect is. (f) Heatmap displayed the correlation between the four m6AIGs and the clinical characteristics and the risk score level

The univariate Cox regression analysis showed that age, stage, T-stage, N-stage, risk score level and the classification were closely correlated with the prognosis (Figure 6(a)). The multivariate Cox regression analysis identified the risk score level (*P* = 0.0111, HR: 2.1238, 95 CI%: 1.1879−3.7969) as independent prognostic factor (Figure 6(b)). A nomogram in the light of age, T-N stage, risk score level and the classification for predicting 3- and 5-year survival was constructed (Figure 6(c)) and calibrated (Figure 6(d-e)), showing the model on 3-year and 5-year prognostic prediction was satisfactory, with C-index of 0.694 (95%CI: 0.620–0.768). The *P* value of likelihood ratio test for C-index was 0.001. The association of the four m6AIGs with the age, gender, stage, T-N-M stage, risk score level and survival state was illustrated in figure 6(f).

### Signaling pathways for risk level subgroups based on GSEA

**Figure 7.**
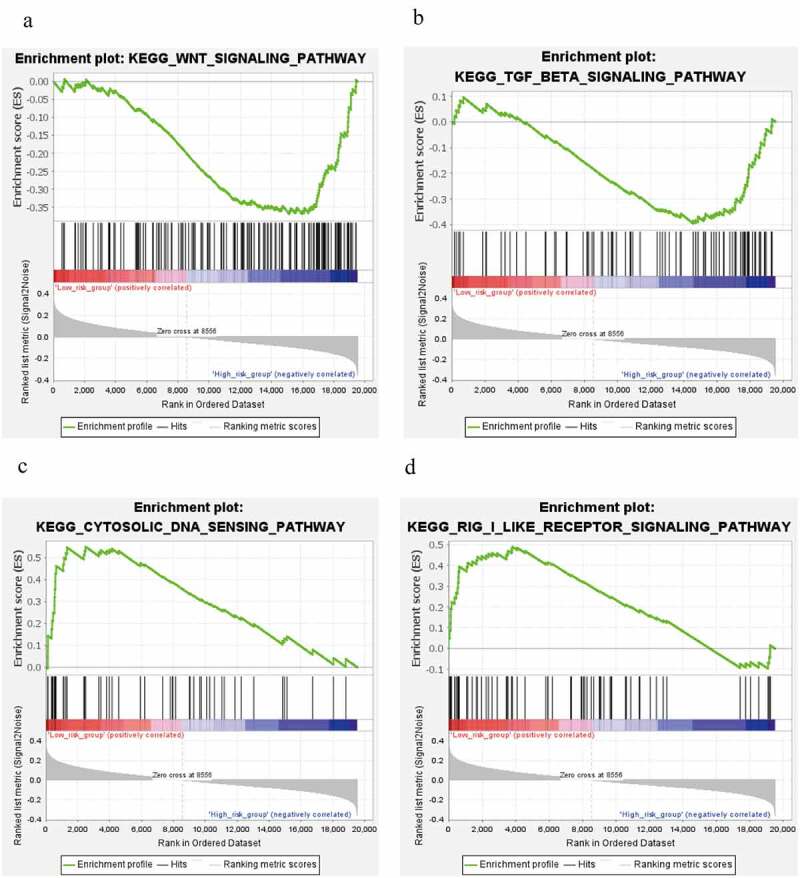
Signaling pathways analysis. (a, b) WNT signaling pathway and TGF-beta signaling pathway in high risk level group. (c, d) cytosolic DNA-Sensing cGAS-STING signaling pathway and RIG-I-like receptors signaling pathway in low risk level group

Signaling pathways with | NES | > 1, NOM *p*-value < 0.05, FDR q-value < 0.25 were considered as statistically significant. GSEA analysis for risk subgroups showed WNT signaling pathway and TGF-beta signaling pathway were involved in regulation of BC progression in the high risk level group (Figure 7(a-b)). In the low risk level group, cytosolic DNA-Sensing cGAS-STING signaling pathway and RIG-I-like receptors signaling pathway were found to be correlated with BC development (Figure 7(c-d)).

## Discussion

It is universally recognized that CD8 + T cells play the role of killing tumor cells by distinguishing tumor-specific antigens presented on major histocompatibility complex class I (MHCI). The function and activation of CD8 + T cells are influenced by cytokines secreted from tumor cells and other cells [29]. The mature and activated infiltrating CD8 + T cells in TME contributed to prolonging the overall survival of patients with malignancies [8,29]. CD4 + T cells discerned antigens derived from major histocompatibility complex class II (MHCII). CD4 + T cells subsets acting as effectors (such as helper T cells and assist cytotoxic T cells) could transition to the memory state after elimination of antigens and cytokines [30,31]. Macrophages are the most faithful partners in tumor growth and metastasis which cause chronic inflammation, facilitate angiogenesis, degrade and remodeling matrix, and assist in tissue invasion and intravasation [32]. Ali et al. [33] showed the proportions of M0, M1, and M2 macrophages possessed prognosis significance according to the clustering analysis and clinical results determined by the proportion of M0 and M2 macrophages [34]. Neutrophils are the first leukocyte to reach the site of inflammation, which can induce tumorigenesis by destroying specific tissues, releasing reactive oxygen species (ROS), and reactive nitrogen species (RNS) or proteases [35,36]. Neutrophils are also attributable to extracellular matrix degradation, tumor cell migration and invasion, and angiogenesis and regulation and suppression of T cells give rise to tumor growth and metastasis [37]. The functional consistency between neutrophils and macrophages is manifested in the synergistic interaction of TME to promote tumor progression and metastasis, which indicates that there are many factors in microenvironment jointly maintaining tumor characteristics, with the same clinical outcomes in BC [38]. Dendritic cells linking the innate and adaptive immune responses activate CD4+ and CD8 + T cells by presenting the specific-antigens, to play the indirect role in anti-tumor immunity; however, the process may be lost or attenuated in TME [39]. Therefore, these immune cells have prominent therapeutic and prognostic values for the final survival of BC patients.

As the most common modification in eukaryotic cells, m6A post-transcriptional modification influences RNA transcription, processing, splicing, degradation and translation; and dysregulated m6A modification in these bioprocesses was highly linked to tumorigenesis [40]. EMT process was facilitated by stable ZMYM1 through up-regulation of METTL3 in gastric cancer [41]. m6A modification of oncogenes of CDCP1 and MYC were elevated due to up-regulation of METTL3, leading to BC for proliferation and progression [42,43]. Therefore, identifying m6A-related genes are crucial for surveillance and management for multiple cancers. In our study, four m6AIGs in BC were screened. KM survival analysis is a univariate analysis, which is easily affected by confounding factors and cannot accurately reflect the prognosis of factors. The multivariate Cox regression analysis is able to exclude the influence. Although KM survival analysis did not show the differential survival for the four m6AIGs, the result from multivariate Cox regression analysis demonstrated BGN, GRK5, IL32 and SREBF1 were independent prognostic factors. Therefore, it is reasonable to believe they were prognosis-associated m6A-related immune genes. The immune infiltration analysis showed they may modulate tumor behaviors by regulating various immunocytes infiltration in microenvironment. TN stage correlation analysis demonstrated BGN, GRK5 and SREBF1 were closely correlated tumor progression. In order to predict prognosis accurately, a nomogram based on age, T stage, N stage, risk score level and cluster was developed, and calibration curve and C-index showed it was a reliable model. Overexpressed BGN facilitates epithelial-mesenchymal transition (EMT) and is significantly linked to the poor prognosis of BC patients [44]. In gastric cancer, BGN promotes tumor angiogenesis through activation of VEGF regulated by TLR signaling pathway, resulting in tumor invasion and progression [45]. Given the positive association of BGN expression with infiltrating CD4 + T cells, Macrophages and Dendritic cells in our study, BGN with m6A modification might regulate the bioprocess of Macrophages to affect the carcinogenesis and progression of BC. GRK5 was found to facilitate proliferation and progression and be related with the regulation of cell cycle in non-small-cell lung cancer [46]. m6A-related GRK5 was downregulated expression in BC and linked to unfavorable prognosis, therefore, we hypothesized downregulated GRK5 chiefly hampered the infiltration of CD8 + T cells, CD4 + T cells and Dendritic cells to attenuate the response of antitumor. Then, modulated by AKT, β-catenin and HIF-1α signaling pathways, IL32 was closely associated with metastasis of gastric cancer [47]. Overexpressed IL32 led favorable prognosis in BC; thereby, we suppose the overexpression of IL32 with m6A modification would boost the recruitment of CD4 + T cells and Dendritic cells, to play the role of antitumor. SREBF1 was involved in fatty acid metabolism, and expression of SREBF1 mediated by AR/mTOR complex accelerated metabolism of fatty acid, to meet the demand for prostate cancer cell growth [48]. In our study, we found the high expression of SREBF1 was correlated with low infiltration level of CD4 + T cell and Dendritic cell, bringing poor prognosis. These findings on the signatures of the four m6AIGs provided theoretical bases for future research.

However, some limitations exist in our study. Firstly, genes with m6A modification in RMVar database are updated continuously, and only the existing genes with m6A modification in database are retrieved in our study. Secondly, the clinical characteristics included in the nomogram for prediction are limited. Thirdly, genetic/epigenetic regulations related to infiltrating immune cells have not been fully studied in BC, so potential regulatory signaling pathways for the four m6AIGs in immune cells are required to be investigated.

## Conclusion

In the study, m6AIGs associated with the prognosis in BC were screened, the correlation of m6AIGs with the infiltration of immune cell and the TNM stage was examined, the BC samples were classified in the light of the four m6AIGs, combined the four m6AIGs with clinical characteristics to analyze the prognosis and the potential regulatory pathways for risk level subgroups were investigated. These works are conductive to identification of immunomarkers with m6A modification in BC.

## Supplementary Material

Supplemental Material

## Data Availability

The data used and analyzed during the present study are available from TCGA (https://portal.gdc.cancer.gov/), GEO (https://www.ncbi.nlm.nih.gov/geo/query/acc.cgi?acc=GSE31684), the RMVar (http://rmvar.renlab.org/index.html) and the InnateDB (https://www.innatedb.com/), the Tumor IMmune Estimation Resource (TIMER, https://cistrome.shinyapps.io/timer/).

## Abbreviation

BC: bladder cancer; m6A: N6-methyladenosine; m6AIGs: m6A-related immune genes; TCGA: The Cancer Genome Atlas; DEGs: differentially expressed genes; PCA: principal component analysis; TME: tumor microenvironment; TIICs: tumor infiltrating immune cells; LASSO: Least Absolute Shrinkage and Selection Operator; KM: Kaplan–Meier; C-index: the concordance index; ROC: receiver operating characteristic; GSEA: Gene Set Enrichment Analysis.
