# Supplementary material for: Signatures and Prognostic Values of N6-methyladenosine (m6A) - related Immune Genes in Bladder Cancer
Source: Bioengineered. 2021 Jun 11;12(1):2649–63. doi: 10.1080/21655979.2021.1937910 (PMC8806594; doi:10.1080/21655979.2021.1937910)
Supplement: Supplemental Material [file KBIE_A_1937910_SM1652.zip › supplementary/SFigure1.docx]

Supplementary Figure 1A


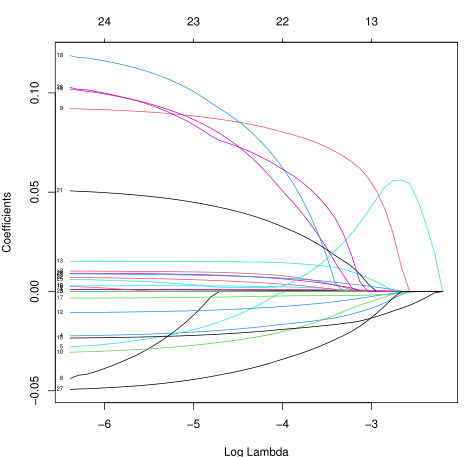


Supplementary Figure 1B


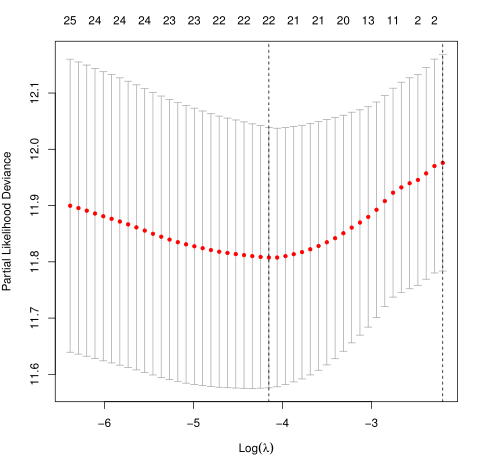


Supplementary Figure 1: LASSO analysis determined the minimum m^6^AIGs for the multivariate Cox regression analysis.
