# Supplementary material for: Signatures and Prognostic Values of N6-methyladenosine (m6A) - related Immune Genes in Bladder Cancer
Source: Bioengineered. 2021 Jun 11;12(1):2649–63. doi: 10.1080/21655979.2021.1937910 (PMC8806594; doi:10.1080/21655979.2021.1937910)
Supplement: Supplemental Material [file KBIE_A_1937910_SM1652.zip › supplementary/STable 2.docx]

Supplementary Table 2: The outcome of the univariate Cox regression analysis

Id Hazard Ration P value

ACAP1 0.8978 (0.8151-0.9888) 0.0287

BCL10 1.0057(0.9712-1.0413) 0.7510

BGN 1.0008(1.0003-1.0013) 0.0016

BIRC5 1.0124(1.0001-1.0249) 0.0488

BST2 0.9990(0.9981-1.0000) 0.0414

C1R 1.0010(0.9982-1.0038) 0.4816

C1S 1.0012(0.9981-1.0044) 0.4458

C3 1.0003(0.9980-1.0026) 0.7949

C7 1.0014(0.9844−1.0187) 0.8722

CAMK2A 1.6584(0.8970−3.0664) 0.1067

CASP6 0.9607(0.9343−0.9877) 0.0046

CCNA2 1.0135(0.9968−1.0305) 0.1143

CCR4 0.9857(0.8401−1.1565) 0.8600

CEBPD 0.9967(0.9915−1.0019) 0.2168

CMA1 1.0091(0.9800−1.0390) 0.5442

COLEC12 1.0405(1.0055−1.0767) 0.0229

CTSG 1.0087(0.9939−1.0237) 0.2497

CXCL11 0.9933(0.9864−1.0001) 0.0551

CXCL12 1.0119(1.0065−1.0173) <0.0001

DDX60L 0.9539(0.8959−1.0157) 0.1404

DHCR24 1.0017(1.0000−1.0033) 0.0474

EGR1 1.0016(0.9996−1.0035) 0.1168

ELANE 1.4240(0.8002−2.5342) 0.2294

FCGR1A 1.0132(0.9419−1.0898) 0.7255

FCN1 1.0217(0.9208−1.1337) 0.6856

GLI1 1.2314(1.0219−1.4839) 0.0287

GRK5 1.1095(1.0707−1.1497) <0.0001

HMGB2 0.9986(0.9945−1.0026) 0.4859

HMGB3 1.0015(0.9923−1.0108) 0.7487

IFI27 0.9992(0.9976−1.0008) 0.3164

IFI6 0.9999(0.9996−1.0003) 0.7331

IFIH1 0.9701(0.9461−0.9946) 0.0173

IFIT1 0.9917(0.9785−1.0050) 0.2183

IFIT3 0.9931(0.9870−0.9992) 0.0265

IL32 0.9920(0.9850−0.9990) 0.0248

IL6ST 1.0129(0.9993−1.0267) 0.0630

IRF4 0.8500(0.7066−1.0226) 0.0849

ITGA3 0.9973(0.9940−1.0007) 0.1159

JUN 1.0017(0.9989−1.0045) 0.2382

KITLG 1.0301(1.0117−1.0487) 0.0012

LRRFIP1 0.9906(0.9576−1.0247) 0.5833

MASP1 1.0249(0.7749−1.3555) 0.8630

MID2 1.1581(1.0143−1.3224) 0.0300

MST1R 0.9597(0.9379−0.9819) 0.0004

MX1 0.9954(0.9883−1.0026) 0.2101

NCKAP1L 1.0029(0.9632−1.0443) 0.8879

NFATC2 1.0818(1.0100−1.1587) 0.0249

NLRP7 0.9986(0.9930−1.0042) 0.6277

OAS1 0.9839(0.9751−0.9928) 0.0004

OAS3 0.9910(0.9805−1.0015) 0.0940

PELI2 1.0998(0.9942−1.2167) 0.0647

PLK1 1.0103(0.9934−1.0274) 0.2349

PPARGC1A 1.0574(0.8616−1.2978) 0.5932

PTCH1 1.2469(1.0504−1.4801) 0.0117

PTGS2 1.0027(0.9965−1.0089) 0.3901

PTX3 1.0072(1.0015−1.0129) 0.0128

RCAN1 1.0237(1.0058−1.0420) 0.0094

RNASE7 1.0069(0.9902−1.0238) 0.4224

RPS6KA5 0.8082(0.5107−1.2790) 0.3633

RSAD2 0.9873(0.9708−1.0040) 0.1358

SELE 0.9897(0.9710−1.0089) 0.2910

SKP2 1.0027(0.9876−1.0181) 0.7245

SLAMF8 0.9842(0.9591−1.0100) 0.2285

SLC22A3 1.0645(1.0108−1.1210) 0.0180

SMAD7 1.0058(0.9740−1.0386) 0.7247

SOCS3 1.0013(0.9981−1.0046) 0.4260

SPHK1 1.0125(1.0004−1.0248) 0.0431

SPP1 1.0001(1.0000−1.0002) 0.0174

SREBF1 1.0064(1.0002−1.0126) 0.0442

STAT5B 1.0006(0.9520−1.0516) 0.9821

TCF4 1.1529(1.0772−1.2338) <0.0001

TNFAIP3 0.9927(0.9823−1.0032) 0.1751

TNFRSF13B 0.8118(0.5934−1.1107) 0.1924

TP73 1.0242(0.9575−1.0955) 0.4870

TPSB2 1.0081(0.9924−1.0240) 0.3148

TRAIP 0.9707(0.8975−1.0498) 0.4566

TREM1 1.0030(0.9751−1.0316) 0.8359

TRIM24 0.9984(0.9821−1.0151) 0.8533

TRIM63 1.0327(0.8735−1.2209) 0.7063

USP2 1.0180(0.9395−1.1032) 0.6626

WDR34 1.0028(0.9973−1.0083) 0.3223

WDR62 1.0859(0.9761−1.2081) 0.1298

WNT2B 1.5173(1.0852−2.1216) 0.0148

YJEFN3 0.9209(0.8583−0.9880) 0.0217

ZBTB16 0.9923(0.7699−1.2791) 0.9527

P<0.05 is statistically significant.
