# Supplementary material for: Signatures and Prognostic Values of N6-methyladenosine (m6A) - related Immune Genes in Bladder Cancer
Source: Bioengineered. 2021 Jun 11;12(1):2649–63. doi: 10.1080/21655979.2021.1937910 (PMC8806594; doi:10.1080/21655979.2021.1937910)
Supplement: Supplemental Material [file KBIE_A_1937910_SM1652.zip › supplementary/STable1.docx]

Supplemental Table 1: Clinical Characteristics

Characteristics Total Percentage

sample size 373

Age

<50 22 5.9%

> =50 351 94.1%

Gender

female 98 26.3%

male 275 73.7%

Stage

Ⅰ 2 0.5%

Ⅱ 103 27.6%

III 140 37.5%

IV 128 34.4%

T

T1 3 0.8%

T2 117 31.4%

T3 195 52.3%

T4 58 15.5%

N

N0 222 59.5%

N1 45 12.1%

N2 74 19.8%

N3 6 1.6%

NX 26 7%

M

M0 179 48%

M1 8 2.1%

MX 186 49.9%
